# Supplementary material for: Anatomical Evaluation of Rat and Mouse Simulators for Laboratory Animal Science Courses
Source: Animals (Basel). 2021 Dec 1;11(12):3432. doi: 10.3390/ani11123432 (PMC8697941; doi:10.3390/ani11123432)
Supplement: Supplementary file 1 [file animals-11-03432-s001.zip › Supplement Materials Table S1.pdf]

SimulRATor

DEUTSCHES ZENTRUM
ZUM SCHUTZ VON
VERSUCHSTIEREN

Freie Universität Berlin

Important Note on Data Protection

Your participation in the survey is voluntary. The survey data will be stored and administered anonymously. It is not possible to draw any conclusions about your participation. All data is managed in strict compliance with current data protection guidelines. Only project members have access to the data and are bound to secrecy.
Under no circumstances will data be passed on to third parties. The data will only be used for research purposes. You can cancel the survey at any time without giving reasons.

SimulRATor-Project:

Anatomic Evaluation – Questionnaire A

ID: ____________

Rat simulator A

**1. How realistic are the following characteristics of rat simulator A in comparison to a live rat?**							
**Characteristics**	**Very realistic** 1	**Quite realistic** 2	**Rather realistic** 3	**Rather unrealistic** 4	**Quite unrealistic** 5	**Very unrealistic** 6	**Not applicable** -99
Overall appearance	O	O	O	O	O	O	O
Haptics	O	O	O	O	O	O	O
Mobility of the skin on the neck	O	O	O	O	O	O	O
Mobility of the skin on the flank	O	O	O	O	O	O	O
Skin texture	O	O	O	O	O	O	O
Body size	O	O	O	O	O	O	O
Body weight	O	O	O	O	O	O	O
Body shape	O	O	O	O	O	O	O
Proportions	O	O	O	O	O	O	O

Characteristics	Very realistic 1	Quite realistic 2	Rather realistic 3	Rather unrealistic 4	Quite unrealistic 5	Very unrealistic 6	Not applicable -99
Gender-specific characteristics	o	o	o	o	o	o	o
Blood vessels	o	o	o	o	o	o	o
Position of the blood vessels	o	o	o	o	o	o	o
Course of the blood vessels	o	o	o	o	o	o	o
Consistency of the blood vessels	o	o	o	o	o	o	o
Mobility of joints	o	o	o	o	o	o	o

2. How realistic are the following characteristics on the head of the rat simulator A in comparison to a live rat?

Characteristics	Very realistic 1	Quite realistic 2	Rather realistic 3	Rather unrealistic 4	Quite unrealistic 5	Very unrealistic 6	Not applicable -99
Overall appearance of the head	o	o	o	o	o	o	o
Head shape	o	o	o	o	o	o	o
Head proportions	o	o	o	o	o	o	o
Mobility of the head	o	o	o	o	o	o	o
Mobility of the lower jaw	o	o	o	o	o	o	o
Degree of mouth opening	o	o	o	o	o	o	o
Appearance of the mouth opening	o	o	o	o	o	o	o
Pharynx and larynx region	o	o	o	o	o	o	o

Characteristics	Very realistic 1	Quite realistic 2	Rather realistic 3	Rather unrealistic 4	Quite unrealistic 5	Very unrealistic 6	Not applicable -99
Teeth	O	O	O	O	O	O	O
Tongue	O	O	O	O	O	O	O
Eyes	O	O	O	O	O	O	O
Ears	O	O	O	O	O	O	O

3. How realistic are the following characteristics on <u>the tail</u> of the Rat simulator A in comparison to a live rat?

Characteristics	Very realistic 1	Quite realistic 2	Rather realistic 3	Rather unrealistic 4	Quite unrealistic 5	Very unrealistic 6	Not applicable -99
Overall appearance of the tail	O	O	O	O	O	O	O
Haptic of the tail	O	O	O	O	O	O	O
Mobility of the tail	O	O	O	O	O	O	O
Skin texture	O	O	O	O	O	O	O
Length of the tail	O	O	O	O	O	O	O
Connection to the trunk / torso	O	O	O	O	O	O	O
Position of the tail blood vessels	O	O	O	O	O	O	O
Course of the tail's blood vessels	O	O	O	O	O	O	O
Quality of the tail's blood vessels	O	O	O	O	O	O	O
Size of the tail's blood vessels	O	O	O	O	O	O	O
Visibility of the blood vessels through the skin	O	O	O	O	O	O	O

**4. How realistic are the following characteristics on the limbs of rat simulator A in comparison to a live rat?**							
**Characteristics**	**Very realistic** **1**	**Quite realistic** **2**	**Rather realistic** **3**	**Rather unrealistic** **4**	**Quite unrealistic** **5**	**Very unrealistic** **6**	**Not applicable** **-99**
Overall appearance of the limbs	O	O	O	O	O	O	O
Haptic of the limbs	O	O	O	O	O	O	O
Proportions of the limbs	O	O	O	O	O	O	O
Posture of the limbs	O	O	O	O	O	O	O
Mobility of the limbs	O	O	O	O	O	O	O
Length of the limbs	O	O	O	O	O	O	O
Toes on the limbs	O	O	O	O	O	O	O

5. From an anatomical point of view, what did you particularly like about rat simulator A?

6. From an anatomical point of view, what did you particularly dislike about rat simulator A?

7. What would you <u>improve</u> from an anatomical point of view in rat simulator A?
