# Supplementary material for: Anatomical Evaluation of Rat and Mouse Simulators for Laboratory Animal Science Courses
Source: Animals (Basel). 2021 Dec 1;11(12):3432. doi: 10.3390/ani11123432 (PMC8697941; doi:10.3390/ani11123432)
Supplement: Supplementary file 1 [file animals-11-03432-s001.zip › Supplement Materials Table S3.pdf]

Important Note on Data Protection

Your participation in the survey is voluntary. The survey data will be stored and administered anonymously. It is not possible to draw any conclusions about your participation. All data is managed in strict compliance with current data protection guidelines. Only project members have access to the data and are bound to secrecy.
Under no circumstances will data be passed on to third parties. The data will only be used for research purposes. You can cancel the survey at any time without giving reasons.

SimulRATor-Projekt:

Anatomic Evaluation – Questionnaire B

ID: ____________

Rat simulator A

Rat simulator B

Rat simulator C

Rat simulator D

Rat simulator E

ID: _____

Please rank the rat simulators based on their overall anatomical characteristics from „most realistic“ to „least realistic“. To do so, please enter the simulator name and its corresponding number as listed above.		
**Ranking**	**Name of the simulator**	**Number**
**Most realistic**		
↓		
↓		
↓		
**Least realistic**		

Do you have comments, praise or criticism? We would be happy about your feedback!

Thank you for your participation!

The SimulRATor-Team

web: www.simulrator.de mail: kontakt@simulrator.de

SimulRATor

ID: _____
