# Supplementary material for: Anatomical Evaluation of Rat and Mouse Simulators for Laboratory Animal Science Courses
Source: Animals (Basel). 2021 Dec 1;11(12):3432. doi: 10.3390/ani11123432 (PMC8697941; doi:10.3390/ani11123432)
Supplement: Supplementary file 1 [file animals-11-03432-s001.zip › Supplementary Materials.pdf]

Table S1. Questionnaire A with the example of rat simulator A**.**

PDF file of questionnaire A. The illustrations were subsequently omitted in the PDF file. For illustrations of the simulators, please see Table 1 or Figures 1–6.

Table S2. The 6–point Likert scale used in questionnaire A. A low rating for a simulator indicates a higher level of realism.

Very realistic	Quite realistic	Rather realistic	Rather unrealistic	Quite unrealistic	Very unrealistic	Not applicable
1	2	3	4	5	6	7

Table S3. Questionnaire B.

PDF file of questionnaire B. The illustrations were subsequently omitted in the PDF file. For illustrations of the simulators, please see Table 1 or Figures 1–6.

Table S4. Ranking table used in questionnaire B.

Ranking	Simulator Name	Number
**Most realistic**		
↓		
↓		
↓		
**Least realistic**		

Table S5. Statistical comparison between rat simulator A and all other rat simulators.

**General Exterior; $p < 0.001$**			
**Simulator 1**	**Simulator 2**	Mean difference	Significance
Rat B	Rat A	0.286	0.888
Rat C	Rat A	2.286*	**<0.001**
Rat D	Rat A	2.000*	**<0.001**
Rat E	Rat A	0.714	0.237
**Tail; $p < 0.001$**			
**Simulator 1**	**Simulator 2**	Mean difference	Significance
Rat B	Rat A	0.357	0.786
Rat C	Rat A	1.967	**<0.001**
Rat D	Rat A	2.500	**<0.001**
Rat E	Rat A	−0.071	0.999

Limb; $p = 0.005$			
**Simulator 1**	**Simulator 2**	Mean difference	Significance
Rat B	Rat A	0.500	>0.999
Rat C	Rat A	1.643	**0.006**
Rat D	Rat A	0.571	>0.999
Rat E	Rat A	−0.010	>0.999
**Head; $p < 0.001$**			
**Simulator 1**	**Simulator 2**	Mean difference	Significance
Rat B	Rat A	0.571	**0.432**
Rat C	Rat A	2.571	**<0.001**
Rat D	Rat A	1.500	**0.002**
Rat E	Rat A	0.500	0.550

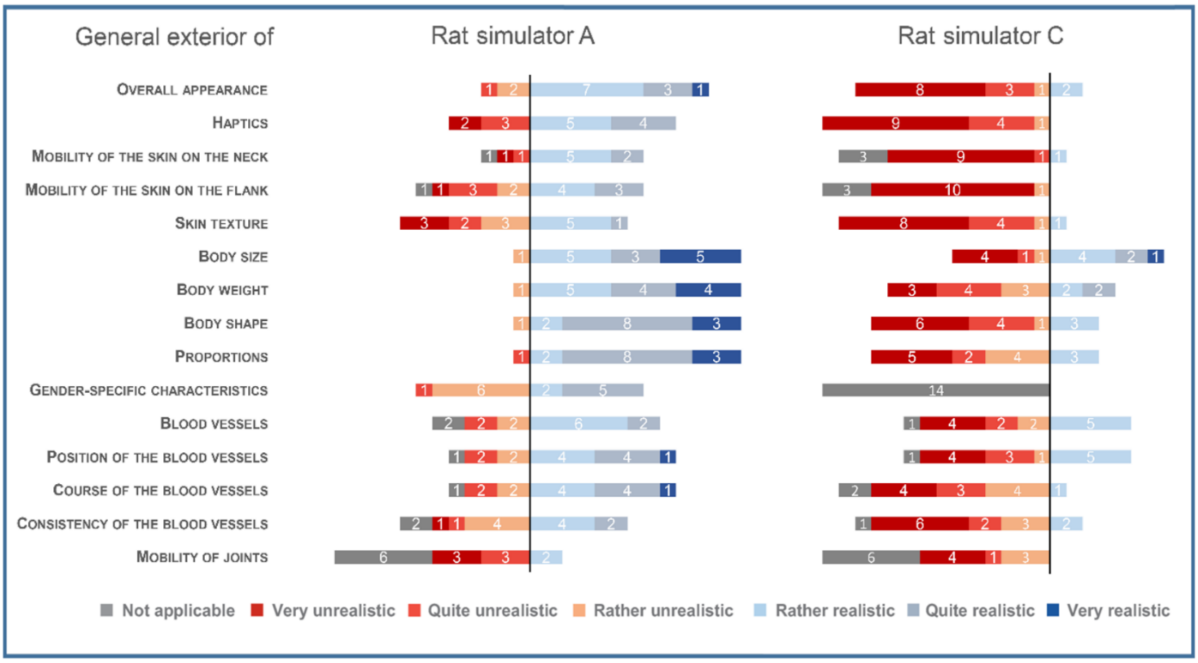

Figure S1. Diverging stacked bar charts comparing the answers of evaluators concerning the general exterior of the lowest (Rat simulator A) and highest rated simulator (Rat simulator C). Values left of the vertical black line are considered "unrealistic", values right of the line are considered "realistic". The white numbers correspond to the response frequency.

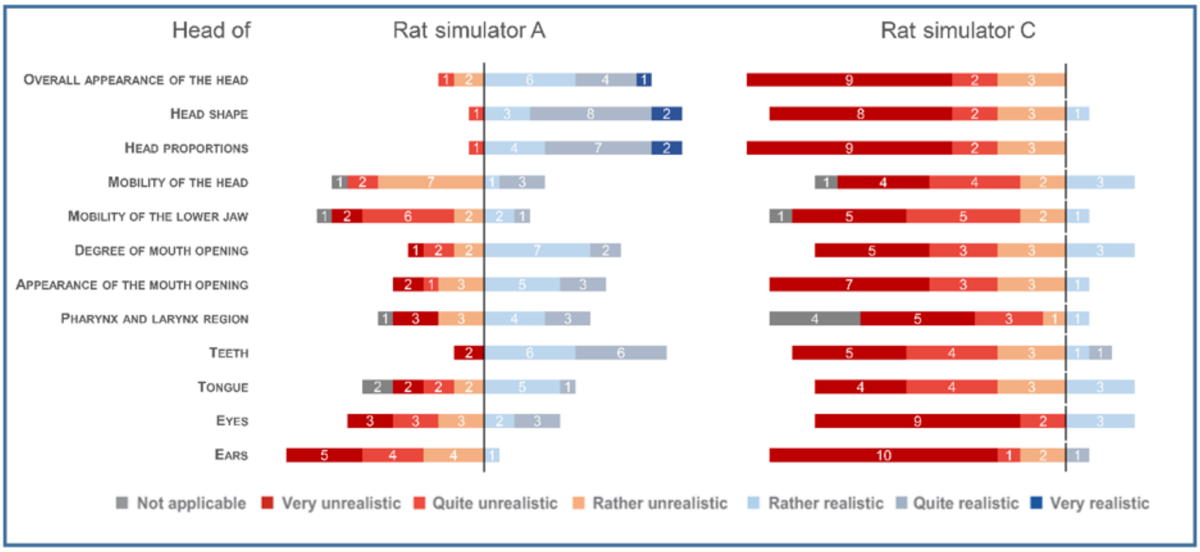

Figure S2. Diverging stacked bar charts comparing the answers of evaluators concerning the head of the second lowest (Rat simulator A) and highest rated simulator (Rat simulator C). Values left of the vertical black line are considered "unrealistic", values right of the line are considered "realistic". The white numbers correspond to the response frequency.

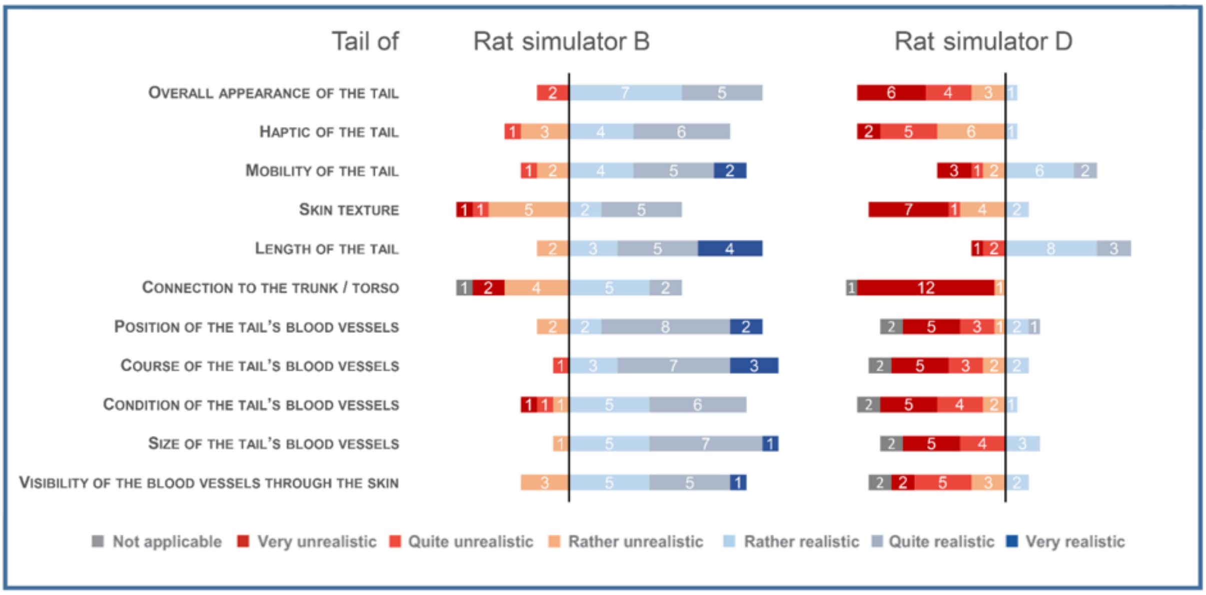

Figure S3. Diverging stacked bar charts comparing the answers of evaluators concerning the tail of the lowest (Rat simulator B) and highest rated simulator (Rat simulator D). Values left of the vertical black line are considered "unrealistic", values right of the line are considered "realistic". The white numbers correspond to the response frequency.

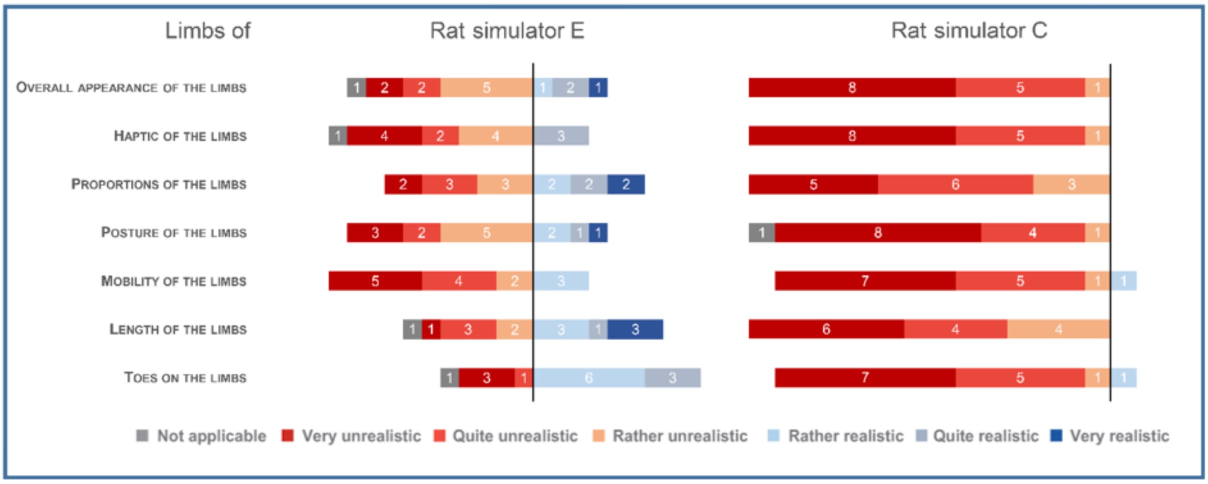

Figure S4. Diverging stacked bar charts comparing the answers of evaluators concerning the limbs of the lowest (Rat simulator E) and highest rated simulator (Rat simulator C). Values left of the vertical black line are considered “unrealistic”, values right of the line are considered “realistic”. The white numbers correspond to the response frequency.
